# Immediate response to major incidents: defining an immediate responder!

**DOI:** 10.1007/s00068-019-01133-1

**Published:** 2019-04-05

**Authors:** Amir Khorram-Manesh, Patricia Plegas, Åsa Högstedt, Mahmoudreza Peyravi, Eric Carlström

**Affiliations:** 1 Department of Surgery, Institute of Clinical Sciences, Sahlgrenska Academy, University of Gothenburg, Gothenburg, Sweden; 2 Emergency and Disaster Medicine, Faculty of Medicine, Shiraz University of Medical Sciences, Shiraz, Iran; 3 Unit of Prehospital Dispatching Center, Region Västra Götaland, Gothenburg, Sweden; 4 Health and Crisis Management and Policy, Sahlgrenska Academy, University of Gothenburg, Gothenburg, Sweden; 5 USN School of Business, University of South-Eastern Norway, Vestfold, Norway

**Keywords:** Immediate responder, First responder, Mass casualty, Management, Public education

## Abstract

**Purpose:**

There is a gap in time between the occurrence of a mass casualty incident (MCI) and the arrival of the first responders to the scene, which offers an opportunity for the public (immediate responders) to perform life-saving measures. The purpose of this study was to identify these measures and the public’s willingness to conduct them.

**Method:**

An extensive literature review was performed to identify the possible measures that can be undertaken by the public. A group of experts were asked to prioritize and rank the feasibility of performing the measures by the public. Finally, the public was asked whether they were willing to do the chosen measures before and after an appropriate education.

**Results:**

Twenty different measures were identified and presented in a questionnaire as statements, which were prioritized and ranked by the expert group into four categories: what (1) should be done, (2) is good to know how, (3) is not necessary to know, and (4) should not be done. All statements were converted into understandable statements and were sent to the public. There were some differences and some agreements between the experts and the public regarding what an immediate responder should do. However, the willingness of the public to perform most of the measures was high and increased after being offered an appropriate education.

**Conclusion:**

The use of immediate responders is a life-saving approach in MCIs and in situations when every minute counts and every human resource is an invaluable asset. Multiple steps, such as education, empowerment, and access, should be taken into consideration to enable bystanders to effectively help struggling survivors.

**Electronic supplementary material:**

The online version of this article (10.1007/s00068-019-01133-1) contains supplementary material, which is available to authorized users.

## Introduction

An increasing number of mass casualty incidents (MCIs) in recent years has revealed some major shortcomings in the medical and non-medical aspects of its management [1–4]. Besides natural disasters and armed conflicts, terrorism and mass shootings have emerged as main causes of mass injuries, deaths, and global threats [5–7]. While Asian and African countries have been the main target for terrorism-related incidents, other countries have not been spared, with the highest number in the UK (*n* = 104), followed by Norway (*n* = 69), USA (*n* = 61), Germany (*n* = 41), France (*n* = 26), and Sweden (*n* = 16) [8–12]. Mass shooting, defined as a shooting where more than four people are involved, has impacted American soil with the highest number of incidents in the past decades. However, other countries have not been untouched, with high morbidity and mortality in France (2015), Norway (2011), and Germany (2009) [8, 13, 14].

Improving the pre-hospital and hospital care can reduce the mortality and morbidity caused by MCIs [5]. For the emergency medical services (EMS) and first responders, the time between the incident and when the victims may be available for treatment (response time) is critical. Any increase in response time due to lack of resources, inadequate infrastructure, etc. will worsen the medical outcome [15–18]. Although most EMS try to respond quickly, a response time of about 10–20 min can often be registered depending on the size of the city, its infrastructure, and traffic situation [19]. This waiting period leaves a gap in time, a critical therapeutic window, where victims cannot receive proper care while waiting for the EMS [15, 20, 21]. Recent MCIs in Paris and Boston enhanced the critical discussion about what could be done at the prehospital level to increase the preparedness for future MCIs [22–26]. Measures such as using Tourniquets to stop hemorrhages were recommended. Nevertheless, a recent study reported that proper capability of conducting these recommendations is lacking in many EU countries [2].

Many video reports from real incidents show the will and availability of the public to help the victims immediately, before any first responder appears on the scene. The question raised has been whether it is possible and what are the requirements to use these “immediate responders” while waiting for the professionals [18, 19, 24–29]? In the WHO report from 2007, “mass casualty management systems” strategies and guidelines, the importance of “a culture of preparedness” at the community level is emphasized. Based on these strategies and guidelines, the passive attitude toward responding to emergencies and MCIs and the expectation of someone else’s responsibility to act needs to change, and civilian preparedness should be included in the national emergency preparedness, globally [1, 29]. Since civilians’ contribution could change the outcome of an accident, hence the number of lives saved, initiatives have been taken to engage the public in the management of MCIs. The American College of Surgeons introduced “the Hartford Consensus” in 2013. Their guidelines aim to create a national policy to enhance survivability from intentional mass casualties and active shooter events (stop bleeding) [27, 30–33]. Similarly, a UK charity, “citizenAID,” aims to empower civilians to act in life-threatening situations before the EMS arrives [34]. These two initiatives together with the WHO guidelines may suggest that civilians could be a good source of primary help at the scene of an incident [27, 30–34]. The task should not necessarily be any kind of treatment; rather, the knowledge of not worsening some critical injuries can also be life-saving. Civilian intervention in the management of airways, stabilizing fractures and the spine, neurovascular assessment, and basic treatment of shock due to bleeding have all been discussed [35–39]. Other organizational aspects of MCI management such as finding a proper place for the deceased and those injured, ambulance parking, and helicopter landing areas, as well as evacuating uninjured people from the incident area, may be other measures that can be done by the public to ease the EMSs work.

Life-saving cardio pulmonary resuscitation (CPR) has successfully been conducted by the public after proper education. Unfortunately, despite given recommendations, proper civilian preparedness for MCIs is lacking. The current limitation in resources and time suggests a need for new approaches [40]. Educational initiatives raising awareness and knowledge of the public to act in difficult situations such as MCIs could increase the survival rate of victims [41, 42].

The aims of this study were to

- identify and suggest which measures can be conducted by the public, hereinafter called “immediate responders” at the prehospital level by asking a group of experts.
- to investigate the willingness of the public to perform various measures before and after an educational initiative.

## Method and material

### Literature review and creating the question set

An extensive literature review was conducted by the main author (AK) and a set of questions, based on the literature recommendations, was retrieved. The following search words were used alone or in combination: mass casualty management, immediate responders, treatment of victims, assessment of victims, educational initiatives, public willingness, expert opinions, and prehospital care, among publications between 2000 and 2018. PubMed, Google Scholar, and Scopus were used as the main search engines. Another author (EC) reviewed and completed the set of questions. All questions were reviewed and approved by all the authors.

Each question was formulated as a statement, which could be answered using a Likert scale from 1 to 7, where 7 meant complete agreement and 1 meant complete disagreement. The number of statements was limited to 20 to enable a high response rate.

### Expert opinion through the questionnaire

The final questionnaire was created by the main author (AK) and thereafter sent to an academic and professional expert group (Appendix 1 in ESM). 14 experts within the relevant fields (three surgeons, three anesthesiologists, four emergency physicians, and four internal medicine specialists) received questionnaire I. 13 experts (93% response rate) replied. The questionnaire was reviewed. Its dimensions and questions were adjusted and clarified. This review was based on a combination of logic, relevance, comprehension, legibility, clarity, and usability. In this way, all statements were tested on their feasibility. All data were collected and reviewed. Simple statistical analysis was used whenever necessary.

### Public opinion through the questionnaire

After finishing the experts’ round, a new questionnaire (no. II) was created for the public with the same statements. However, medical words were translated into simple daily words to enable full understanding of the context for ordinary people. A self-selection web questionnaire with the same questions and statements as the one sent to the expert group was prepared by one of the authors (PP), using Google forms (Appendix 2 in ESM). All the 20 statements were closed ended and were reviewed by all the authors. Each statement had (a) and (b) sections, where (a) featured “what would you be willing to do now” and (b) “what do you think you would be willing to do if you received an appropriate education first.” In an information section preceding the statement, it was stated that education in the text refers to education for civilians, which currently is not available, and its content not yet decided. This allowed us to investigate the willingness of participants to act on the scene of an emergency. It was mandatory to answer all the statements on a Likert scale from 1 to 7 (as mentioned before), and it was not possible to skip any statement. The respondents could leave a comment after answering all the statements, when they also reviewed their age, gender, occupation, and eventual activity in a voluntary organization. Completion of the questionnaire took around 10 min.

### Population

People between 15 and 75 years old, living in Sweden, referred to here as civilians, were included. People under 15 years of age and over 75 were excluded. Depending on their occupations, and eventual activity in voluntary organizations, the respondents were divided into two groups: “medical knowledge” (MK) and “no medical knowledge” (NMK). The MK group was divided into three subgroups: (1) registered healthcare personnel: doctors and registered nurses, (2) people with health care education but not registered as healthcare personnel: assistant nurses, students in healthcare professions, military, police officers, and firefighters, and (3) people active in voluntary health-related organizations such as the Red Cross, SMS Lifesaver, Swedish Lifesavers, etc. The NMK group consisted of people who might be active in non-health-related voluntary organizations with no acquired medical knowledge at all, e.g., the scouts.

### The statistics, data collection, and analysis

Power calculation resulted in a need for 200 respondents (statistical power of 0.80, the medium effect size of 0.3, and *α* significance level of 0.05). The questionnaire was sent out digitally using self-selection and was distributed via email and social media mostly using Facebook, where it was shared widely. The questionnaire was also sent out with the monthly email from the Swedish Red Cross foundation. All data were collected and transferred to an excel file.

#### Experts data

The answers given by the experts were converted into a table, and the mean point for each question was calculated.

#### Public data

To ensure anonymous participation, each respondent was assigned an ID based on the time they answered. All data obtained were controlled after the end of the survey and coded in the statistical program, SPSS. The final data were thereafter analyzed using the same program. Cronbach alpha was conducted to evaluate the reliability of the survey. The main part of the statistical analyses was the descriptive data.

To simplify the results of the questionnaire, all statements were grouped by their relevance into three categories by the working group;

- The treatment category consisted of statements about acting or “treating” a patient: simple life-sustaining actions, CPR, to manage a drowning accident, stop bleeding, use aid to control hemorrhages, stabilize fractures, stabilize the neck and lower back, and implementing a cervical collar.
- The assessment category consisted of statements about “assessing” an injury or situation: shock assessment, neurovascular assessment, fracture positioning, triage, vital indication for intervention, and evacuating or barricading.
- Finally, the organization and logistics category included statements about the organization and logistic around accidents, including laws and regulations regarding MCIs and disasters; acting against a perpetrator; knowledge about hot, warm, and cold zones; organizing the scene of the accident; knowledge about high-risk accidents; and securing the scene of the accident.

In each of the three categories, the mean, median, and standard deviation (SD) were calculated for each question and in total, both for all respondents and for the NMK group. In the NMK group, calculations were done to see whether individuals went from being negative (Likert scale 1–3, or neutral: Likert scale 4, on the a) alternative “what are you willing to do now”) to being positive (Likert scale 5–7 on the b) alternative “after an appropriate education” in the same statements; calculations were made in the different categories. The McNemar–Bowker test of symmetry was used for comparison and for calculating a *P* value. Statistical significance was defined as *P* < 0.05, and 95% confidence intervals were obtained, when necessary.

Cronbach’s alpha was used to measure the reliability or internal consistency of the questionnaire. The Cronbach’s Alpha, measured for our questionnaire, was 0.954. Other statistical figures are presented as mean, median, and SD. Significance was calculated as mentioned in the method section.

### Ethical considerations

The participants received written information about the study. The information included the study’s purpose and stated that participation was voluntary. Participants were informed that they were free to withdraw at any time. They were assured of strict confidentiality and secure data storage. It complied with the ethical principles stipulated by Swedish law (SFS 2008:192). In Sweden, ethical approval is mandatory if the research includes: sensitive data on the participants such as race, ethnical heritage, political views, religion, sexual habits, and health or physical interventions or uses a method that aims to affect the person physically or psychologically (SFS 2003:460). This study, however, was exempt from ethics approval requirements as it did not fulfill any of these aspects and was based on questionnaire data from individuals who freely contributed with their views.

## Results

### The literature review and the set of questions

The following results were obtained from the literature search (2000–2018). The number of hits was reduced by adding new search words from 124,000 hits using mass casualty management, to immediate responders (24,500 hits), treatment of victims (18,500 hits), assessment of victims (18,300 hits), educational initiatives (10,900 hits), public willingness (3780 hits), expert opinions (3500 hits), and prehospital care (443).

All 443 papers were checked for their relevance to the topic. Duplicates, papers not related to the topic, summaries, and non-scientific papers were excluded. The remaining articles were studied thoroughly and the information was included in the paper (references).

### The response from experts

Table 1 shows the responses from the expert group. There was a difference between various specialist groups and within one specialist group. Questions 1–5, 7, 11, 14, 16, and 18 were favorable measures chosen by the experts. These questions dealt with life-saving measures, CPR, understanding shock, drowning, control of hemorrhages, knowing the principals of neck and spine stabilization, knowledge on evacuation and barricading, and knowledge of risk with various scenarios. Questions 6, 10, 12, 13, and 19, 20 were less interesting and were borderline measures (using a tourniquet, performing triage, acting on a vital indication, using a neck collar, securing the incident site, and knowing the related disaster and MCI laws and rules). Finally, the most controversial questions were 8, 9, 15, and 17, which dealt with assessment of the distal status of a fracture, reposition of a fracture, combating a preparator, and knowledge of organizing and safeguarding the incident site (see Appendix one in ESM). One interesting observation was the high points given by anesthesiologists to four questions: Q7 (fracture stabilization), Q10 (performing triage), Q12 (acting on vital indication), and Q16 (knowledge on different risks and workings zones of an incident). By dividing the answers in the Likert scale into negative (1-3), neutral (4), and positive (5–7) answers, the mean of all the results for each question determined whether the measure could be recommended or not. Measures with a mean over 3.5 were understood as recommended by the expert. The working group analyzed the results and decided to divide all the measures into four distinct groups as follows:

**Table 1:** The expert groups’ responses to the questionnaire

	EP	Med	Med	Med	EP	Med	EP	EP	Surg	Surg	Surg	Anest	Anest	Mean
Q1	7	6	6	7	5	6	7	6	6	6	4	5	7	5.86
Q2	7	6	6	7	5	6	6	6	6	6	4	5	7	5.82
Q3	4	3	4	7	3	3	5	5	2	3	4	3	6	4.69
Q4	5	4	6	7	6	2	4	7	5	6	5	4	7	5.32
Q5	7	5	6	7	6	5	6	6	6	4	6	6	7	5.65
Q6	1	4	3	4	1	1	5	6	2	5	3	2	5	3.32
Q7	1	4	3	4	1	1	2	4	2	3	3	4	5	4.48
Q8	1	4	3	4	1	1	1	4	1	3	2	1	3	2.13
Q9	1	4	2	4	1	1	1	4	1	2	1	1	3	1.86
Q10	1	3	4	2	1	1	3	5	1	2	4	2	7	3.11
Q11	3	3	6	5	1	1	4	5	1	3	5	4	6	3.83
Q12	2	6	3	5	2	1	3	5	1	3	3	3	7	3.57
Q13	1	5	4	5	1	1	3	5	1	3	5	1	5	3.04
Q14	4	5	5	4	1	5	5	6	4	5	5	6	7	4.26
Q15	2	5	3	4	1	1	4	5	1	4	3	1	2	2.39
Q16	1	4	5	2	1	1	5	1	1	3	4	1	5	3.75
Q17	1	2	4	2	1	1	5	5	1	3	2	1	5	2.54
Q18	3	4	6	6	2	4	5	6	3	4	4	5	5	4.39
Q19	1	3	6	6	2	3	5	6	1	2	4	2	6	3.44
Q20	2	2	5	5	1	1	5	7	1	2	5	3	4	3.06

Questions 1–5, 7, 11, 14, 16, and 18 seem to be favorable measures chosen by the experts. Questions 6, 10, 12, 13, and 19, 20 are less favorable, while the most controversial questions are 8, 9, 15, and 17 (see results for more details)

- Should be able to conduct life-saving measures, CPR, control of hemorrhages by compression, know how to act in cases of drowning, know how to evacuate and barricade, and know the risks involved in various threats.
- Is Good to know referred to how to simply handle a shock, use a tourniquet.
- Not necessary to know how to stabilize fractures, triage, stabilize the spine, act on a vital indication, use a neck collar, self-defense, organize and safeguard the incident site, and learn about emergency laws and rules.
- Should Not assess the distal status of a fracture and conduct fracture repositioning.

### Responses from the public

Of the 1246 respondents, 12 were not registered correctly in the web-questionnaire, leaving 1234 respondents who were included in the study. Around 62% (*n* = 759) were female and 38% (*n* = 475) male. There was a higher representation of females within all the groups. The age distribution had a culmination at 26–30 years of age. Around 76% of the respondents were working (*n* = 934), 16% (*n* = 198) were students, 5% (*n* = 59) were retired, 2% (*n* = 24) were unemployed, and 2% (*n* = 19) had other activities. When specifying their occupation, the respondents filled in over 200 different occupations.

The share of the MK group was 45% (*n* = 558) of the total number of respondents; 91 registered healthcare professionals (doctors and registered nurses), and 467 with prior healthcare education, but not registered. The remaining 251 respondents were active in healthcare-related voluntary organizations. The willingness to respond in the MK group was much higher than in the NMK group, in both alternatives of before (73.3% vs. 45.3%) and after education (87% vs. 81%). However, the difference in the number of people changing their attitudes from negative or neutral into positive after education was not significant statistically. Since the focus of this study was on the civilians with no medical background, no further results will be presented for the MK group.

The NMK group consisted of 676 respondents (55%) with no medical knowledge. Their occupations varied from teachers, administration and communication workers, engineers, students, to retired individuals. As mentioned before, the results obtained from this group were divided into three categories: treatment, assessment, and organization and logistics. In general, all participants showed a willingness to act on the scene, although they had no knowledge. This willingness increased significantly when they were offered an educational initiative.

- Treatment category had the highest number of positive responses overall for both all respondents and the NMK group. A total of 72% (all) and 61% (NMK), respectively, were willing to act initially on the scene. Their willingness increased to 91% and 89%, respectively, after an appropriate education. Among the statements, willingness to perform simple life-sustaining actions increased from 92 to 97% in all respondents and 87–96% in the NMK group, before and after the education. The same results were obtained about performing CPR (91–97%, and 84–97%, respectively) and control of hemorrhages (92–95%, and 86–94%, respectively). Statements about fracture stabilization (35% vs. 52%), management of spine (27% vs. 47%), and use of cervical collar (40% vs. 55%) had the lowest figures for all respondents and in the NMK group (Table 2). After an appropriate education, many respondents with negative or neutral attitudes moved to the group with a positive attitude. The highest move was seen in the task dealing with simple life-sustaining actions, while stabilizing the neck and lower back had the lowest move with 71%. The difference between the statements before and after the education was statistically significant (Table 3).

**Table 2:** Distribution of dimensions within the NMK group and all the respondents, in the treatment category

Treatment	Negative 1–3	Neutral 4	Positive 5–7	Mean = *M*	Median ~ *X*	SD
Simple life-sustaining actions	6% (4%)	7% (4%)	87% (92%)	6.04 (6.37)	7.0 (7.0)	1.38 (1.17)
After education	2% (2%)	2% (1%)	96% (97%)	6.62 (6.7)	7.0 (7.0)	0.95 (0.91)
CPR	9% (5%)	7% (4%)	84% (91%)	5.95 (6.31)	7.0 (7.0)	1.46 (1.26)
After education	1% (2%)	2% (1%)	97% (97%)	6.66 (6.74)	7.0 (7.0)	0.88 (0.84)
Drowning accident	24% (16%)	17% (13%)	59% (71%)	4.88 (5.38)	5.0 (6.0)	1.86 (1.78)
After education	7% (6%)	4% (4%)	89% (90%)	6.18 (6.27)	7.0 (7.0)	1.42 (1.42)
Stop bleeding	6% (3%)	8% (5%)	86% (92%)	6.02 (6.36)	7.0 (7.0)	1.38 (1.17)
After education	3% (3%)	3% (2%)	94% (95%)	6.58 (6.69)	7.0 (7.0)	1.14 (0.10)
Use aid to stop bleeding	18% (10%)	14% (11%)	68% (79%)	5.19 (5.75)	5.0 (7.0)	1.72 (1.60)
After education	6% (4%)	3% (2%)	91% (94%)	6.39 (6.52)	7.0 (7.0)	1.34 (1.18)
Stabilize fractures	48% (34%)	17% (14%)	35% (52%)	3.73 (4.51)	4.0 (5.0)	2.00 (2.09)
After education	9% (8%)	7% (6%)	84% (86%)	5.89 (6.06)	7.0 (7.0)	1.63 (1.60)
Stabilize neck and lower back	60% (42%)	13% (11%)	27% (47%)	3.18 (4.12)	3.0 (4.0)	2.00 (2.25)
After education	13% (11%)	10% (7%)	77% (82%)	5.54 (5.82)	6.0 (7.0)	1.80 (1.72)
Cervical collar	46% (34%)	14% (11%)	40% (55%)	3.82 (4.54)	4.0 (5.0)	2.04 (2.15)
After education	11% (10%)	7% (5%)	82% (85%)	5.84 (6.03)	7.0 (7.0)	1.73 (1.67)
All	27% (19%)	12% (9%)	61% (72%)	4.85 (5.42)	5 (6.5)	1.73 (1.68)
All after education	6% (6%)	5% (3%)	89% (91%)	6.21 (6.35)	7.0 (7.0)	1.36 (1.18)

The values without parentheses represent the NMK group (*n* = 676), and the values in parentheses represent the results from all the respondents (*n* = 1234)

**Table 3:** Percentages of respondents in the NMK group (*n* = 676) who were negative (1–3) or neutral (4) on the (a) alternative “willing to do now” became positive (5–7) on the (b) alternative, “after education,” on the same statement, regarding the statements in the treatment category

Treatment	Percentages who changed from negative or neutral to positive (%)	*P* value
Simple life-sustaining actions	86	< 0.001
CPR	86	< 0.001
Drowning accident	79	< 0.001
Stop bleeding	74	< 0.001
Use aid to stop bleeding	82	< 0.001
Stabilize fractures	79	< 0.001
Stabilize neck and lower back	71	< 0.001
Cervical collar	74	< 0.001
All	79
- Assessment category consisted of respondents with lower positive responses; initially, 50% of all responders and 34% of the NMK group were positive. The figures increased to 83% and 80%, respectively, after the education. For the statement about neurovascular assessment, only 30% of all respondents and 15% of the NMK group were positive about acting. These figures, however, changed to 76% and 73%, respectively, after an appropriate education. In this group, 43% of the NMK group and 62% of all were positive in assessing shock. The figures changed to 89% and 91%, respectively after an appropriate education. Triage and knowing about the concept of evacuating and barricading had the highest improvement among the statements after an appropriate education, while fracture positioning seems to be a critical and hard maneuver for the public (Table 4). The difference between statements before and after the education was statistically significant (Table 5).

**Table 4:** Distribution of dimensions within the NMK group and all the respondents, in the assessment category

Assessment	Negative 1–3	Neutral 4	Positive 5–7	Mean = *M*	Median ~ *X*	SD
Shock	39% (24%)	18% (14%)	43% (62%)	4.16 (4.98)	4.0 (5.0)	1.87 (1.91)
After education	7% (5%)	4% (4%)	89% (91%)	6.12 (6.29)	7.0 (7.0)	1.42 (1.33)
Neurovascular assessment	73% (55%)	12% (14%)	15% (31%)	2.63 (3.49)	2.0 (3.0)	1.70 (2.01)
After education	16% (14%)	11% (10%)	73% (76%)	5.34 (5.60)	6.0 (6.0)	1.82 (1.79)
Fracture positioning	64% (49%)	12% (11%)	24% (40%)	3.06 (3.83)	3.0 (4.0)	1.91 (2.18)
After education	14% (14%)	11% (8%)	75% (78%)	5.43 (5.63)	6.0 (7.0)	1.80 (1.81)
Triage	40% (27%)	18% (14%)	42% (59%)	4.00 (4.79)	4.0 (5.0)	1.94 (1.97)
After education	11% (10%)	8% (6%)	81% (84%)	5.69 (5.92)	6.0 (7.0)	1.72 (1.64)
Act on vital indication	39% (26%)	16% (13%)	45% (61%)	4.09 (4.84)	4.0 (5.0)	2.00 (2.02)
After education	12% (10%)	12% (9%)	76% (81%)	5.53 (5.80)	6.0 (7.0)	1.71 (1.65)
Evacuate or stay inside	47% (37%)	16% (17%)	37% (46%)	3.76 (4.22)	4.0 (4.0)	2.01 (2.01)
After education	10% (10%)	5% (5%)	85% (85%)	5.89 (5.94)	7.0 (7.0)	1.67 (1.69)
All	51% (36%)	15% (14%)	34% (50%)	3.62 (4.36)	4 (4.5)	1.91 (2.02)
All after education	12% (10%)	8% (7%)	80% (83%)	5.62 (5.86)	6 (7)	1.69 (1.66)

The values without parentheses represent the NMK group (*n* = 676), and the values in parentheses represent the results from all the respondents (*n* = 1234)

**Table 5:** Percentages of respondents in the NMK group (*n* = 676) who were negative (1–3) or neutral (4) on the (a) alternative “willing to do now” became positive (5–7) on the (b) alternative “after education” in the same statement, regarding the statements in the assessment category

Assessment	Percentages who changed from negative or neutral to positive (%)	*P* value
Shock	84	< 0.001
Neurovascular assessment	69	< 0.001
Fracture positioning	69	< 0.001
Triage at mass casualty scenarios	71	< 0.001
Vital indication	64	< 0.001
Evacuate or stay inside	79	< 0.001
All	73
- Organization and logistics category consisted of respondents, of which 52% in total and 41% in the NMK were initially positive about acting. These figures increased to 78% and 74%, respectively, after being offered an education (Table 6). Overall, these statements seem to be frightening for the public. Organizing, securing, and knowledge in law, etc. are not interesting; thus, initially, less than 50% were positive about acting in most of the statements. Statements about: participation in high-risk incidents, getting knowledge in various incident zones, and knowledge about law and rules regarding disasters and emergency incidents were not initially appreciated. However, the participants´ attitudes toward these statements had the highest increase after an appropriate education. The difference between statements before and after an education was statistically significant (Table 7).

**Table 6:** Distribution of dimensions within the NMK group and all the respondents, in the organization and logistic category

Organization and logistics	Negative 1–3	Neutral 4	Positive 5–7	Mean = *M*	Median ~ *X*	SD
Act against a perpetrator	48% (41%)	15% (14%)	37% (45%)	3.72 (4.11)	4.0 (4.0)	2.03 (2.10)
After education	22% (19%)	12% (12%)	66% (69%)	5.03 (5.18)	5.0 (6.0)	1.92 (1.93)
Hot, warm, and cold zones	36% (27%)	17% (13%)	47% (60%)	4.20 (4.79)	4.0 (5.0)	2.02 (2.01)
After education	17% (14%)	11% (9%)	72% (77%)	5.32 (5.54)	6.0 (6.0)	1.88 (1.82)
Organize the incident site	31% (23%)	12% (11%)	57% (66%)	4.63 (5.10)	5.0 (5.0)	2.05 (1.98)
After education	11% (10%)	6% (6%)	83% (84%)	5.85 (5.97)	7.0 (7.0)	1.69 (1.66)
High-risk accidents	52% (40%)	16% (16%)	32% (44%)	3.43 (4.10)	3.0 (4.0)	1.96 (2.12)
After education	22% (19%)	14% (11%)	64% (70%)	4.95 (5.22)	5.0 (6.0)	1.98 (1.97)
Secure incident site	28% (20%)	12% (10%)	60% (70%)	4.69 (5.24)	5.0 (6.0)	1.98 (1.91)
After education	13% (11%)	4% (4%)	83% (85%)	5.82 (5.95)	7.0 (7.0)	1.75 (1.71)
Civil law, rules regarding accidents and disasters	75% (61%)	11% (13%)	14% (26%)	2.47 (3.10)	2.0 (3.0)	1.66 (1.97)
After education	10% (8%)	11% (9%)	79% (83%)	5.73 (5.87)	6.0 (7.0)	1.62 (1.57)
All	45% (35%)	14% (13%)	41% (52%)	3.86 (4.41)	4 (4.5)	1.95 (2.02)
All after education	16% (13%)	10% (9%)	74% (78%)	5.45 (5.62)	6 (6.5)	1.81 (1.78)

The values without parentheses represent the NMK group (*n* = 676), and the values in parentheses represent the results from all the respondents (*n* = 1234)

**Table 7:** Percentages of respondents in the NMK group (*n* = 676) who were negative (1–3) or neutral (4) on the (a) alternative “willing to do now” that became positive (5–7) on the (b) alternative “after education,” in the same statement, regarding the statements in the organization and logistics category

Organization and logistics	Percentages who changed from negative or neutral to positive (%)	*P* value
Act against a perpetrator	52	< 0.001
Hot, warm and cold zones	55	< 0.001
Organize the incident site	69	< 0.001
High-risk accidents	51	< 0.001
Secure the incident site	69	< 0.001
Civil law, rules regarding accidents and disaster	77	< 0.001
All	62

Table 8 shows a comparison between what the expert group recommended and what the public felt comfortable with doing before and after an appropriate education.

**Table 8:** A comparison between what the expert group recommended and what the public (all responders) felt comfortable in doing before and after an education

No.	Task description	EG	PBE (%)	PAE (%)
1	Life-saving actions	SB	92	97
2	CPR	SB	91	97
3	Knowledge, assessment, and management of shock by simple measures	GT	62	91
4	Handling a case of drowning	SB	71	90
5	Stopping a hemorrhage through compression	SB	92	95
6	To assess the need and use a TORNIQUET	GT	79	94
7	To stabilize a fracture	NN	52	86
8	To assess distal status and penetration risk of a fracture	SN	31	76
9	To assess the need for repositioning a fracture	SN	40	78
10	To triage and evacuate low priority cases from the scene	NN	59	84
11	Learn the principals for stabilization of spine	NN	47	82
12	Learn the principles of acting on vital indication despite the risk for morbidities	NN	61	81
13	To handle a cervical collar	NN	55	85
14	Knowledge about barricading and evacuation	SB	46	85
15	Knowledge of techniques in self-defense	NN	45	69
16	To differentiate between strategic, tactical, and operational levels/zones	NN	60	77
17	Knowledge about organizing an incident site	NN	66	84
18	Knowledge about the risks of different incidents	SB	44	70
19	Knowledge about the safety of the scene	NN	70	85
20	Knowledge about civilian and public rights and laws	NN	26	83

The recommendations are given by the expert group as *SB* should be able, *GT* good to know, *NN* not necessary to know, *SN* should not. The public’s responses are given as percent. (*EG* expert group, *PBE* public before education, *PAE* public after education)

## Discussion

The aims of this study were: (a) to identify which measures can be performed by the public at the prehospital level, and thus define the possible tasks of an “immediate responder,” and (b) to investigate the willingness of the public to become an “immediate responder.” our results define an immediate responder and offer a good foundation for a future curriculum to be used for public education. To the best of our knowledge, this is the first survey exploring different professionals’ opinion on whether the public should be active participants in the management of an emergency (except cardiac arrest and drowning), and what they should do if that is possible? It is also the first study to investigate civilians’ willingness to respond to major emergencies and MCIs, regarding treatment, assessment, and organization issues during an emergency.

Around 40% of prehospital deaths due to accidental injury are potentially preventable if, within an acceptable response time, civilians start basic treatment strategies, such as broader first aid and simple airway management. According to the WHO, civilians should be educated in first aid, or other simple procedures needed for quick response to emergencies [43]. The time between the incident and the arrival of first responders is a therapeutic window, offering an opportunity for bystanders to act on the scene and influence the medical outcome of an incident [15, 18, 20–23]. The Hartford Consensus aims to use this therapeutic window to address the number one most preventable cause of death after both military and civilian injuries, i.e., external hemorrhages, using multiple steps, such as education, empowerment, and access [27, 30–33, 44–50]. Others have discussed the overall possibility for bystanders to perform other measures as immediate responders [15, 20, 21, 35, 36]. In the UK-based project citizenAID [34], measures such as initial triage, hemorrhage control, and airway management are suggested as feasible tasks to be performed by civilians, even without previous education.

In concordance with the findings from Ross et al., only 68% of the respondents in our NMK group were initially positive towards controlling hemorrhages in the treatment category. Ross et al. [50] reported that most civilians (64%) were not comfortable with responding to a traumatic medical emergency due to multiple barriers, including feeling inadequately trained. Nevertheless, the figure increased to 91% (vs. 96% in Ross study), when the respondents were offered an appropriate education. An increased willingness to act after education has been reported in other studies such as the one in California by Kano et al. [42], and the Norwegian study by Bakke et al. [35]. These statements support the results and aims of this study that some vital measures can be undertaken by civilians after an appropriate education.

Among recommendations given in the literature, some measures might be performed immediately, without losing time, and before the arrival of the first responders, e.g., triage, using a tourniquet, evacuation and quick transport of victims from the scene, barricading and isolating victims of a mass shooting, etc. [1, 18, 22, 23]. Additionally, our literature review revealed other measures, which were included in our questionnaire as statements and were assessed and approved by our expert group as what an immediate responder in Sweden could be expected to do at the incident site [51–53].

Despite different choices made by the members of our expert group, we could categorize the results into four categories of: what an immediate responder should do, needs to know how to do, does not need to know, and should not know at all. Accordingly, we could also highlight what could be included in a curriculum for immediate responders, should anyone apply for the position. Surprisingly, the Swedish public expressed its readiness to perform all of the suggested measures. Although there are some differences between the experts and the public’s views on feasible measures, the sum of all choices defines what an immediate responder should be able to do (Table 8). These findings are in line with earlier reports, of which some claim much more involvement by the public in the medical management of victims of MCIs [1, 2, 18, 22–26, 28–36]. In this study, the Swedish public expressed a high willingness to get engaged in the treatment of victims by stabilizing fractures, neck and lower back, implementing a cervical collar, and defending against a perpetrator, which are tasks that were not approved by the expert group or is dangerous for their lives. Hence, they were very motivated. This motivation was much higher after being offered an appropriate education. Similar results have been shown in other reports, with a higher rate of motivation in females than in males [41, 42, 54].

With initiatives such as “citizenAID” in the UK, “the Hartford consensus” in the US, and the WHO guidelines and recommendations, our results and desire to strengthen the Swedish public’s role in acting in emergencies and MCIs do not seem to be exaggerated and indicate a need for a new educational initiative [1, 27, 30–34]. Currently, all Swedish school students have the first aid education in their curriculum. Despite the introduction of new courses [55], the current curriculum does not cover trauma and mass casualty situations. The topics included in our questionnaire may constitute a foundation for future national and or international educational initiatives for both adults and youth. Pedagogic professionals and experts within the related fields should gather to create a curriculum that transforms bystanders into active immediate responders and decide upon the length and content of such an educational initiative. From a Swedish perspective, such an education can be given in various stages, offering different levels of knowledge, skills, and competency based on the individual’s willingness, background, and interest. Similar programs have already been suggested [56]. Evaluation of the content and length of one of the “stop the bleed” courses, in San Antonio, Texas, USA in 2017 showed that just a short course, 1 h, is enough for civilians to both feel comfortable in stopping bleedings using tourniquets and performing it correctly, by applying pressure [47–50].

## Limitations

There are some limitations to this study.

1. The literature search included only English articles. Recommendations and inputs in other languages and the personal experience of the working group may have influenced the choice of literature and questions.
2. Secondly, despite the hard work of clarifying all the statements in the simple language, when converting the statements in the experts’ questionnaire to the one for the public, some statements may have been difficult for the public to understand. A face-to-face meeting might have given other results.
3. Third, the distribution of the public questionnaire represents a so-called “virtual snowball sampling.” This type of sampling has many advantages and disadvantages. It helps to identify individuals of interest for this research, allows for the possibility to increase the representativeness of the results, can increase the number of responses and decrease the sampling time, and is cheap. On the other hand, sample selection is biased towards the characteristics of the online population such as gender, age, education level, and socioeconomic belonging [57]. Our sample size has a skew gender and age distribution, with more women and young participants. However, the same results have been obtained by other studies using other types of statistical analysis [54]. Other statistical analyses were used when investigating the public responses. Statistical tests based on variables for age and gender were not made on the public results, due to the focus of this part of the study on the willingness of all civilians as a group. *P* value was calculated with the McNemar–Bowker test of symmetry on the NMK group to investigate how many changed from being negative or neutral toward a measure into being positive after an appropriate education. It can be discussed that such calculations cannot be made since the sample size was not randomized and did not correspond to the population. Having this in mind, the outcome of the test showed that there was a significant difference in all the 20 statements. The McNemar–Bowker test was also used in this study [50], where the participants in the study were also recruited using self-selection and thus not a randomized sample.

## Conclusion

MCIs take lives, which can be saved by having prehospital immediate response. The use of immediate responders before the arrival of first responders is a life-saving approach in situations when every minute counts and every human resource is an invaluable asset. This highlights the need for engagement by the public at incident sites. Multiple measures, such as education, empowerment, and access, should be taken into consideration to enable bystanders to effectively help struggling survivors.

## Electronic supplementary material

Below is the link to the electronic supplementary material.

Supplementary material 1 (DOCX 799 kb)
