# Supplementary material for: Immediate response to major incidents: defining an immediate responder!
Source: Eur J Trauma Emerg Surg. 2019 Apr 5;46(6):1309–20. doi: 10.1007/s00068-019-01133-1 (PMC7691304; doi:10.1007/s00068-019-01133-1)

Appendix

Appendix one: Shows the question set sent to the experts.

1. Should the public be able to conduct some life-saving measures at the scene of an MCI while waiting for the EMS?

Complete agreement 7 6 5 4 3 2 1 Complete disagreement

1. Should the public be able to conduct CPR?

Complete agreement 7 6 5 4 3 2 1 Complete disagreement

1. Should the public be able to understand the mechanism of shock, assess a life-threatening shock and handle it by simple measures?

Complete agreement 7 6 5 4 3 2 1 Complete disagreement

1. Should the public have the knowledge of handling a case of drowning?

Complete agreement 7 6 5 4 3 2 1 Complete disagreement

1. Should the public be able to stop a hemorrhage through compression?

Complete agreement 7 6 5 4 3 2 1 Complete disagreement

1. Should the public be able to assess the need and use a TORNIQUET due to a life-threatening extremity injury?

Complete agreement 7 6 5 4 3 2 1 Complete disagreement

1. Should the public be able to stabilize a fracture?

Complete agreement 7 6 5 4 3 2 1 Complete disagreement

1. Should the public be able to assess distal status and penetration risk of a fracture?

Complete agreement 7 6 5 4 3 2 1 Complete disagreement

1. Should the public be able to assess the need for fracture reposition?

Complete agreement 7 6 5 4 3 2 1 Complete disagreement

1. Should the public be able to Triage, Sieve and Sort during a MCI and evacuate low priority cases from the scene?

Complete agreement 7 6 5 4 3 2 1 Complete disagreement

1. Should the public learn the principals for stabilization of spine?

Complete agreement 7 6 5 4 3 2 1 Complete disagreement

1. Should the public learn the principles of acting on vital indication despite the risk for morbidities?

Complete agreement 7 6 5 4 3 2 1 Complete disagreement

1. Should the public be able to handle a neck collar?

Complete agreement 7 6 5 4 3 2 1 Complete disagreement

1. Should the public have knowledge about barricading and evacuation, e.g., school attacks?

Complete agreement 7 6 5 4 3 2 1 Complete disagreement

1. Should the public have knowledge of techniques in self-defense during ongoing violence, e.g., school attacks?

Complete agreement 7 6 5 4 3 2 1 Complete disagreement

1. Should the public be able to differentiate between strategic, tactical, and operational levels/zones?

Complete agreement 7 6 5 4 3 2 1 Complete disagreement

1. Should the public have knowledge about organizing an incident site, e.g., ambulance parking, casualty collecting areas, etc.?

Complete agreement 7 6 5 4 3 2 1 Complete disagreement

1. Should the public have knowledge about the risks of accidents, explosions, chemical incidents, fire, and terror attacks?

Complete agreement 7 6 5 4 3 2 1 Complete disagreement

1. Should the public have knowledge about how the incident site can be safe and who is responsible?

Complete agreement 7 6 5 4 3 2 1 Complete disagreement

1. Should the public have knowledge about civilian and public rights and laws during MCI management?

Complete agreement 7 6 5 4 3 2 1 Complete disagreement

Appendix two: The public questionnaire


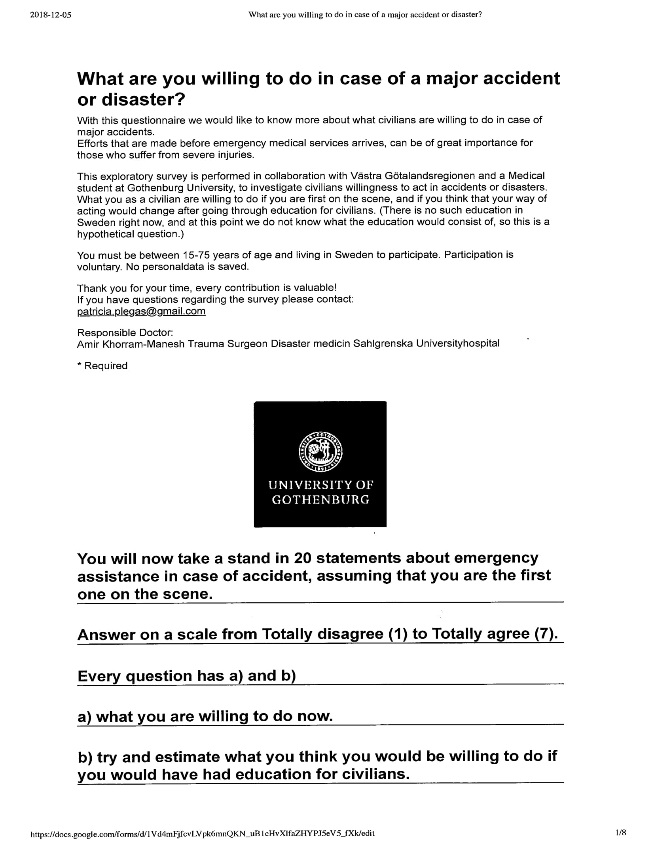

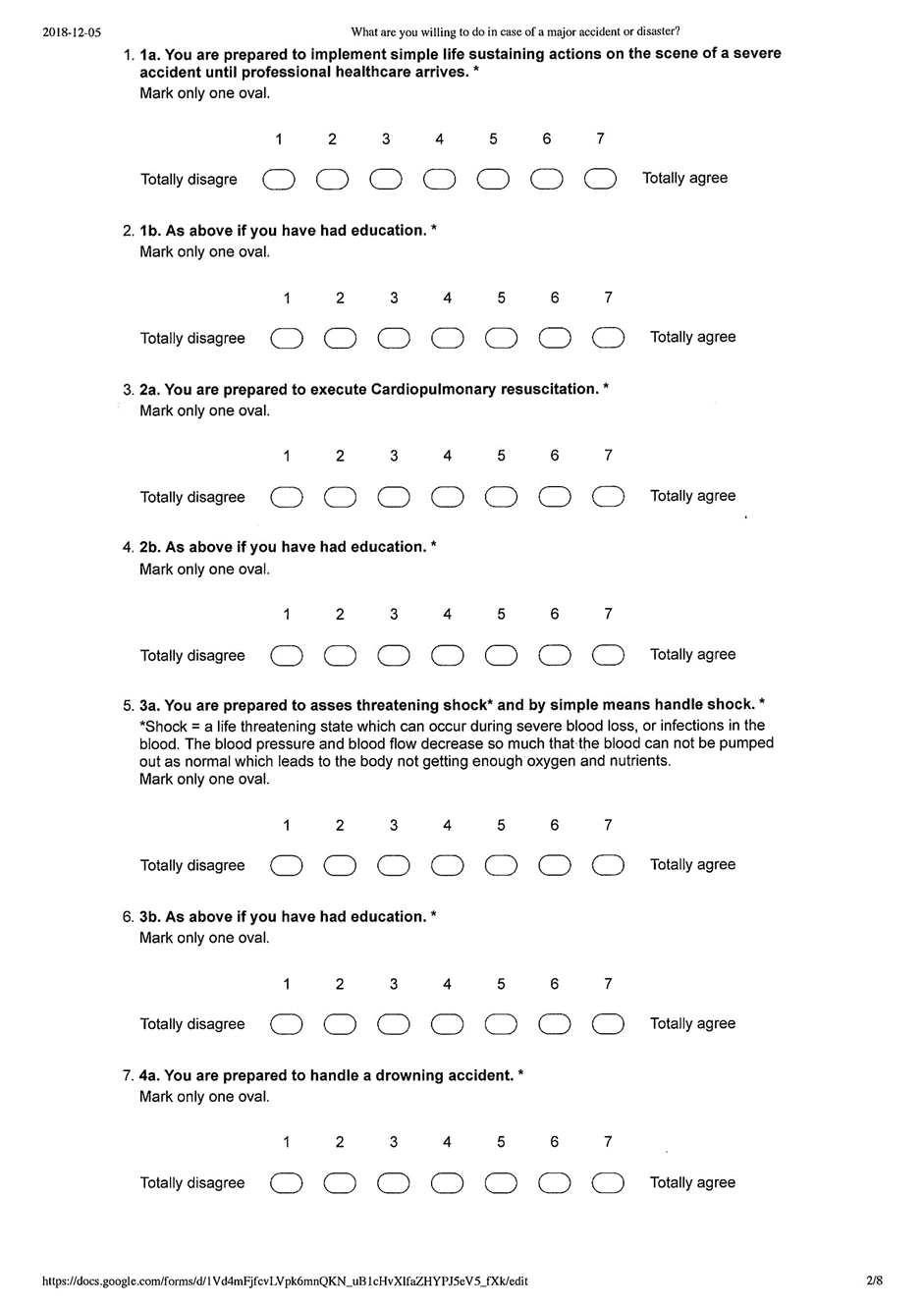

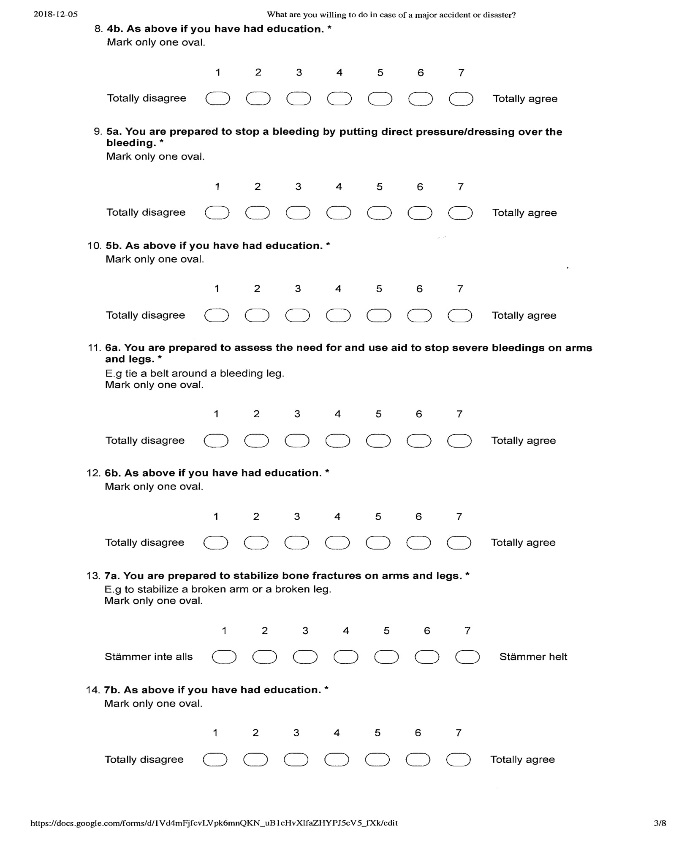

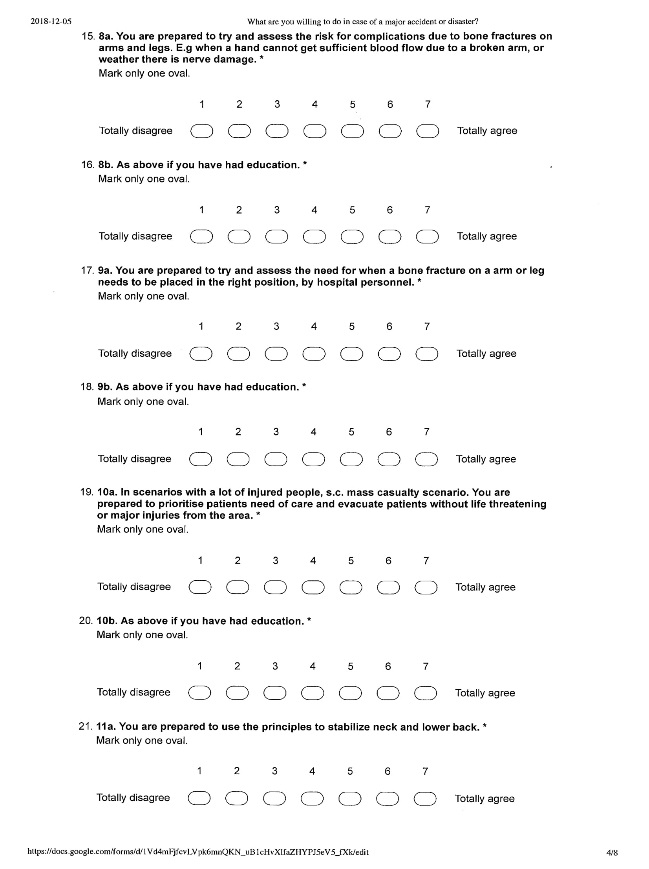


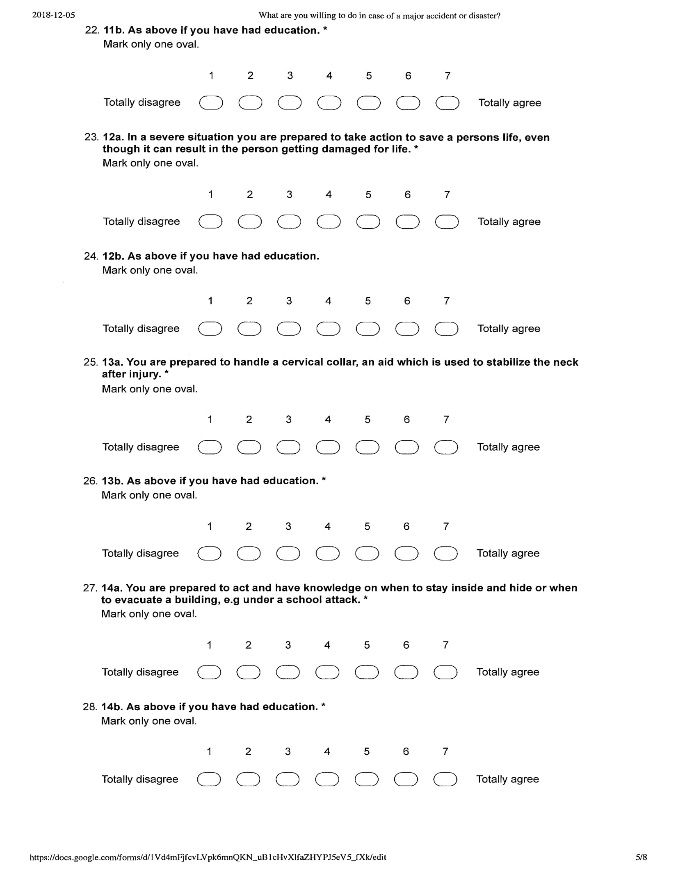

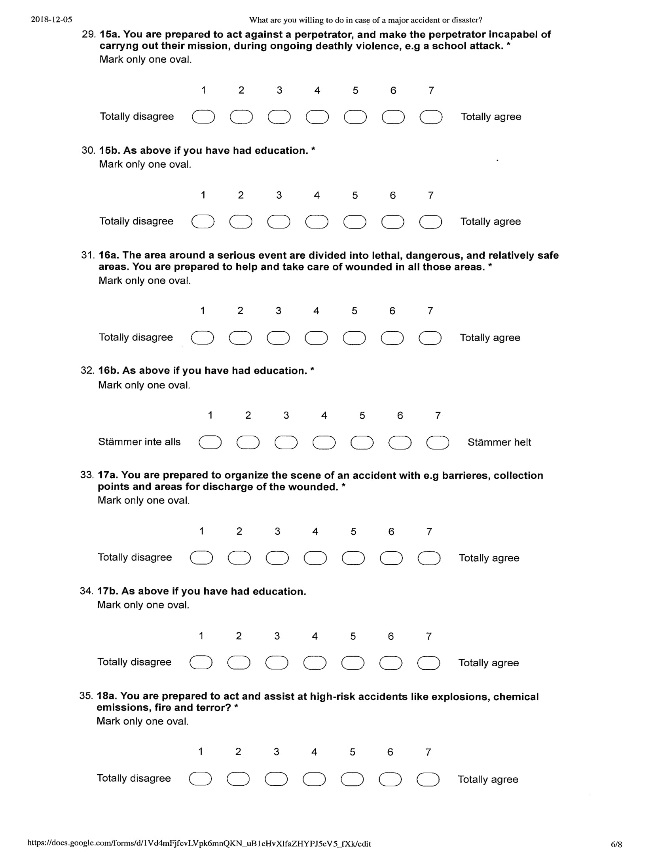

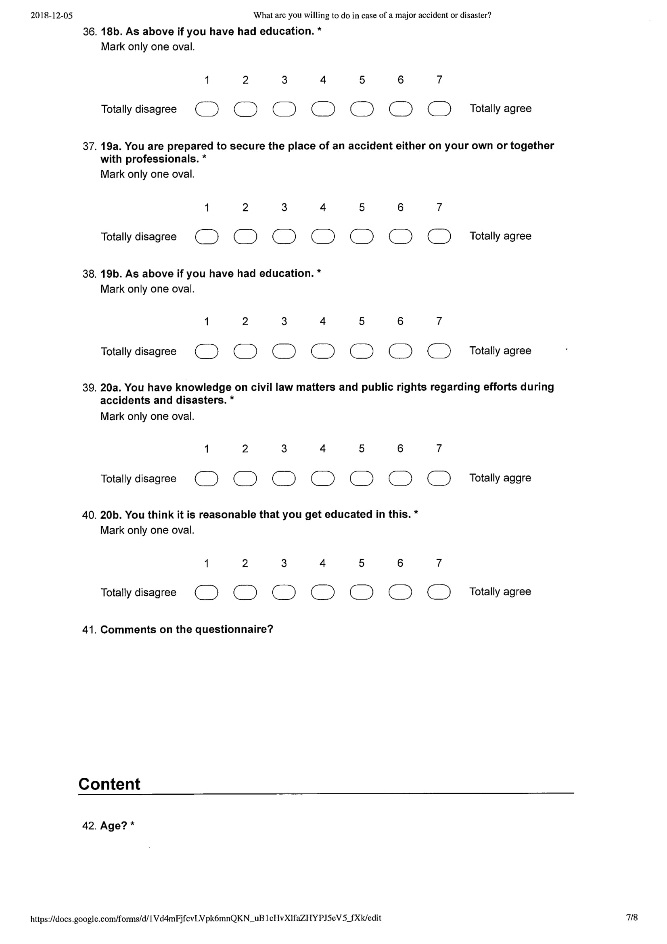

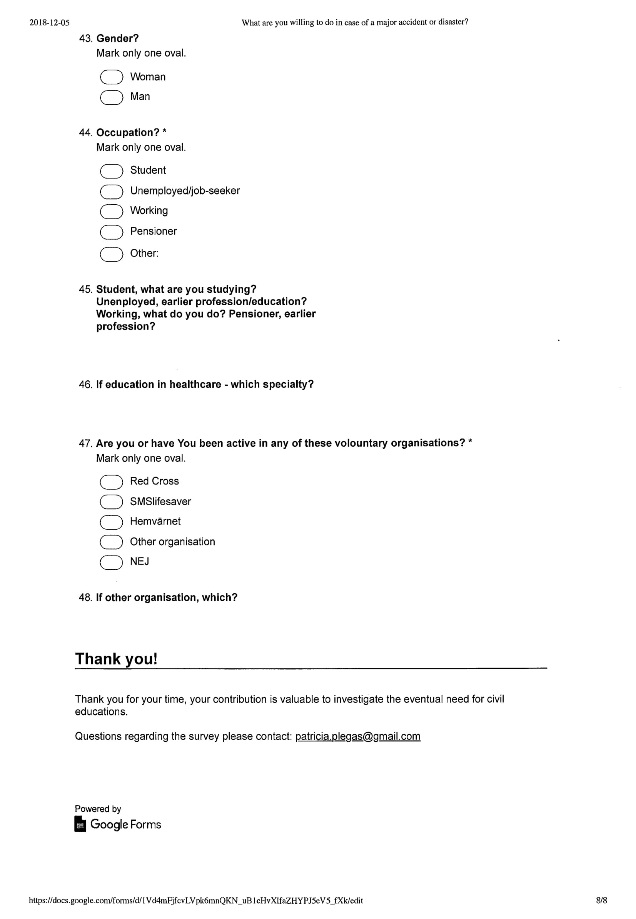

Supplement: Supplementary file 1 — Supplementary material 1 (DOCX 799 kb) [file 68_2019_1133_MOESM1_ESM.docx]
